# Disparities in Peripheral Circulatory Complication‐Related Mortality in Type 2 Diabetes Mellitus Patients: A CDC Analysis (1999–2020)

**DOI:** 10.1002/edm2.70083

**Published:** 2025-07-29

**Authors:** Iqra Shahid, Hadia Ahmad, Saad Ashraf, Qasim Mehmood, Azka Ijaz, Suraksha Rani, Sara Sohail, Shameer Iqbal Ghuman, Mahnoor Fatima, Minaam Farooq, Hafsa Shahid, Minhal Chaudhry, Aayush Chaulagain

**Affiliations:** ^1^ King Edward Medical University Lahore Pakistan; ^2^ Department of Medicine Dow University of Health Sciences Karachi Pakistan; ^3^ Shifa Clinical Research Center, Shifa International Hospital Islamabad Pakistan; ^4^ Department of Medicine King Edward Medical University Lahore Pakistan; ^5^ Department of Medicine Sir Syed College of Medical Sciences for Girls Karachi Pakistan; ^6^ Department of Medicine Brigham and Women's Health Hospital, HIM USA; ^7^ Department of Medicine Patan Academy of Health Sciences Lalitpur Nepal

**Keywords:** CDC WONDER, disparities, mortality, peripheral circulatory complications, type 2 diabetes mellitus

## Abstract

**Introduction:**

Peripheral circulatory complication (PCC), a significant complication of type 2 diabetes mellitus (T2DM), poses a considerable mortality burden in the United States. This study aimed to analyse demographic and geographic disparities in PCC‐related mortality in T2DM patients from 1999 to 2020.

**Methods:**

Utilising the CDC WONDER database, we utilised death certificates to identify PCC‐related deaths using ICD‐10 Code E11.5 and calculated age‐adjusted mortality rates (AAMRs) per 1,000,000 individuals. Joinpoint regression analysis was used to assess annual percent changes (APCs) in mortality rates.

**Results:**

PCC caused 81,793 deaths. The AAMR increased from 1999 to 2004 (APC: 5.88, 95% CI: 2.44 to 12.70), followed by a decrease from 2004 to 2014 (APC: −3.70, 95% CI: −5.83 to −2.45), and an increase again until 2020 (APC: 8.34, 95% CI: 6.05 to 11.50). Males consistently exhibited higher mortality rates (AAMR: 11.08, 95% CI: 11.00 to 11.6) than females (AAMR: 8.34, 95% CI: 8.26 to 8.43). Racial/ethnic disparities were evident, with American Indian or Alaskan natives showing the highest AAMR (19.76) compared to Asian or Pacific Islanders (6.11). Geographic disparities were observed, with the Midwest region (AAMR: 12.86) and West Virginia (AAMR: 18.52) exhibiting significantly higher mortality rates.

**Conclusion:**

Mortality trends associated with PCC in T2DM patients have shown complex trajectories, with notable disparities across demographic and geographic lines. Further research is needed to comprehensively understand the dynamics of PCC and its implications for public health.

## Graphical abstract

This national analysis reveals rising mortality from peripheral circulatory complications in U.S. type 2 diabetes patients from 1999 to 2020, with stark disparities by race, gender, and geography. American Indian/Alaska Natives, males, and rural populations were disproportionately affected, highlighting the urgent need for targeted interventions.

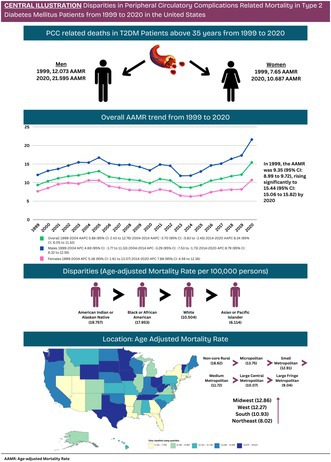

## 1 Introduction

Type 2 diabetes mellitus (T2DM) is a chronic metabolic disorder characterised by insulin resistance, β‐cell dysfunction, and systemic hyperglycemia [1]. Globally, over 537 million adults live with diabetes, a figure projected to rise to 783 million by 2045, driven by urbanisation, sedentary lifestyles, and obesogenic environments [2].

Peripheral circulatory complications (PCC), such as occlusion of the peripheral vessels of the upper and lower limbs, represent one of the most debilitating macrovascular complications of T2DM. Diabetes mellitus (DM) remains a major risk for PCC, with DM patients having more than two‐fold increased prevalence of PCC compared with the general population [3]. The pathophysiology of these complications in T2DM is multifactorial, driven by a confluence of metabolic, inflammatory, and haemodynamic derangements that synergistically impair vascular homeostasis [4]. Central to this process is chronic hyperglycaemia, which activates four major pathways implicated in vascular damage: the polyol pathway, hexosamine pathway, protein kinase C (PKC) activation, and advanced glycation end‐product (AGE) formation. AGEs, formed via non‐enzymatic glycation of proteins and lipids, crosslink with collagen in the vascular basement membrane, reducing arterial elasticity and trapping low‐density lipoprotein (LDL) particles within the subendothelial space, thereby accelerating atherosclerosis [5]. Microvascular dysfunction amplifies ischaemia through capillary rarefaction and basement membrane thickening, which impede oxygen diffusion to peripheral tissues. This is compounded by autonomic neuropathy, which disrupts sympathetic vascular tone, causing arteriovenous shunting and reduced perfusion to the distal extremities [6]. In macrovascular beds, diabetic atherosclerosis is distinct in its morphology: plaques exhibit greater calcification, necrotic core volume, and distal multisegment involvement, particularly in below‐the‐knee arteries, complicating endovascular and surgical revascularization [7].

Nearly 15% of adults in the United States are currently affected by diabetes mellitus [8]. In the U.S., approximately 8.5 million adults with T2DM have PAD, with annual diagnoses increasing by 1.3% since 2015, disproportionately impacting racial minorities and low‐income groups [9, 10] Diabetic foot ulcers (DFUs), a common peripheral complication and a precursor to 80% of diabetes‐related amputations, develop in 15%–25% of T2DM patients [11, 12].

Alarmingly, the 5‐year mortality rate following a DFU diagnosis is 45%–60%, surpassing mortality rates for breast cancer (10%–15%) and colorectal cancer [12]. During their lifetime, DM precedes 80% of non‐traumatic amputations and is associated with a 5‐year mortality rate exceeding 50% [13, 14]. Despite advancements in glycemic control and revascularisation techniques, mortality attributable to peripheral circulatory disease (PCD) remains unacceptably high, exposing profound inequities in care access and outcomes across racial, ethnic, and socioeconomic lines [15].

In the U.S., mortality rates for PCD complications declined by 40% from 1999 to 2009 due to improved glycemic control and smoking cessation efforts but have since plateaued, with a 1.2% annual increase observed from 2010 to 2020, disproportionately affecting marginalised communities [16, 17].

The investigation of geographical disparities is a crucial part of the health system, allowing us to discover information regarding the influence of location on the prevalence of disease. This further helps in the appropriate management of the disease. Geographical disparities in health outcomes have been the subject of extensive investigation within the field of public health, providing valuable insights on disease prevalence and management [18]. There are several studies reporting the variations in rates of diabetes in the United States according to regions [8]. Several studies have emphasised the significant regional variations in diabetes and coronary artery disease (CAD) rates across the United States [18]. Similarly, diabetic complications are not uniformly present. These complications appear disproportionately higher in marginalised populations due to biological susceptibility and structural inequities. Individuals in the lowest income quintile have 3.2‐fold higher mortality rates from PCD complications than those in the highest quintile, reflecting disparities in access to podiatric care (30% vs. 65%) and statin therapy (42% vs. 68%) [19, 20]. Genetic predisposition, variation in lifestyles and disparities in the availability of healthcare may be factors explaining these findings [21].

However, studies focusing solely on circulatory complications of diabetes are lacking. Even though prior studies have focused on inpatient cohorts or single institutions, lacking generalisability, national analyses often aggregate diabetes‐related deaths without disentangling PCD‐specific outcomes. The CDC WONDER database provides a unique platform to address these limitations, offering 21 years of population‐level mortality data (1999–2020) stratified by demographics, geography, and cause of death. This allows us to identify high‐risk sub‐groups and evaluate the impact of policy interventions on mortality trajectories.

Geographic and demographic analysis and variation in trends help in resource allocation, allowing the system to target the areas with the highest needs and severity. In addition to this, gender and racial disparities need to be addressed, as systemic factors and socio‐economic inequalities result in disproportionate access to healthcare [22, 23]. The impact of all of these factors on the mortality trends is imperative to pave the way for an effective development of equitable healthcare interventions. Our study aims at inspecting in detail the various patterns of mortality resulting from peripheral circulatory complications in diabetic patients in the United States over the period 1999–2020. By thoroughly examining regional, gender, and racial disparities, this research seeks to uncover and illuminate the complex patterns of CAD mortality among diabetic populations. The main goal is to provide meaningful insights that lead to the formation of targeted interventions and health policies, ideally helping to reduce disparities and improve outcomes for at‐risk populations.

## 2 Methods

### 2.1 Study Design

Mortality data were obtained from the Centers for Disease Control and Prevention Wide‐Ranging Online Data for Epidemiologic Research (CDC‐WONDER) database [24], a comprehensive repository of death certificate records from all U.S. states and the District of Columbia. Multiple causes of death data were utilised to find death certificates that included any mention of mortality related to peripheral circulatory complications in patients with T2DM. Data extraction was performed utilising the International Classification of Diseases, Tenth Revision (ICD‐10) code E11.5, referring to Type 2 diabetes mellitus with circulatory complications, specifically involving peripheral circulation. Institutional review board (IRB) approval was not needed, as the data were de‐identified and publicly accessible. This study was conducted according to the Strengthening and the Reporting of Observational Studies in Epidemiology (STROBE) guidelines [25].

### 2.2 Data Extraction

Data were stratified by population characteristics, including gender, race, age group, place of death, state, urban–rural classification, and census region. Race/ethnicity was classified as Non‐Hispanic (NH) Asian or Pacific Islander, NH American Indian or Alaska Native, NH White, and NH Black or African American. The place of death was classified into medical facilities, the decedent's residence, nursing homes or long‐term care facilities, hospice centers, and other places. Age groups were divided into 10‐year intervals, ranging from 15 years to 85 years and older. Census regions were defined as the Northeast, South, West, and Midwest. Urban–rural classification followed the 2013 U.S. Census Bureau definition [26], with urban areas defined as having a population of 500,000 or more, and rural areas defined as those with populations below 500,000.

### 2.3 Statistical Analysis

The data were presented as age‐adjusted mortality rates (AAMRs) and crude death rates (CRs). AAMRs were derived by applying age‐specific mortality rates to the 2000 U.S. standard population, producing a weighted average that facilitates equitable evaluations of mortality rates among different populations or time periods [27]. AAMRs were employed to evaluate mortality trends by race/ethnicity, gender, census region, state, and urban–rural classification, while CRs were utilised for age‐group analyses.

We performed trend analyses utilising the Joinpoint regression program version 5.0.2 [28] to estimate annual percentage changes (APCs), with their equivalent 95% confidence intervals (CIs). The Joinpoint software identifies the simplest joinpoint model by starting with the fewest joinpoints and sequentially assessing the statistical significance of adding additional joinpoints up to a predefined maximum, with Monte Carlo permutation tests [29]. In order to determine whether APCs represented increasing or decreasing trends, the slope of mortality rate changes was evaluated for significant deviation from zero using a two‐tailed t‐test. A pairwise comparison between groups was conducted to assess the parallelism of trends. A *p* value of less than 0.05 was regarded as statistically significant.

## 3 Results

### 3.1 Overall

The total death toll due to Peripheral circulatory complications (PCC) in patients with diabetes mellitus in the United States from 1999 to 2020 reached 81,793, indicating a notable burden of this condition (Table 1 and Table S1).

**TABLE 1:** Demographic characteristics of deaths due to peripheral circulatory complications in type 2 diabetes mellitus patients from 1999 to 2020.

Variable	PCC deaths, *n* (%)	AAMRs* (95% CI*) per 100, 000
Overall Population	81 793 (100)	11.08 (11.01–11.16)
Sex		
Male	36 141 (44.1)	14.89 (14.75–15.03)
Female	45 652 (55.8)	8.35 (8.26–8.43)
Census Region		
Northeast	11 716 (14.32)	8.02 (7.88–8.17)
Midwest	21 441 (26.2)	12.86 (12.69–13.03)
South	29 343 (35.9)	10.93 (10.81–11.06)
West	19 293 (23.9)	12.27 (12.09–12.44)
Race/Ethnicity		
American Indian or AlaskaNative	984 (1.2)	19.76 (18.45–21.06)
Asian or Pacific Islander	1671 (2.0)	6.11 (5.82–6.41)
Black or African American	11 873 (14.56)	17.95 (17.62–18.28)
White	67 265 (82.4)	10.50 (10.43–10.58)
Age		
35‐44	443 (0.54)	—
45‐54	2672 (3.27)	—
55‐64	9769 (12.0)	—
65‐74	19 712 (24.1)	—
85+	21 452 (26.23)	—
Urbanization		
Large Central Metropolitan	20 327 (25.0)	10.068 (9.929–10.207)
Large Fringe Metropolitan	15 818 (19.3)	9.048 (8.906–9.19)
Medium Metropolitan	18 418 (22.5)	11.717 (11.547–11.887)
Small Metropolitan	9397 (11.5)	12.906 (12.644–13.168)
Micropolitan	10 023 (12.3)	13.753 (13.483–14.024)
Noncore	7810 (9.5)	13.473 (13.173–13.773)
Place of Death		
Medical facility‐Inpatient	26 752 (32.7)	—
Medical Facility‐Outpatientor ER	4930 (6.02)	—
Medical Facility‐Dead onArrival	357 (0.44)	—
Medical Facility‐StatusUnknown	46 (0.06)	—
Decendent's Home	19 523 (23.9)	—
Hospice Facility	2508 (3.07)	—
Nursing Home/Long TermCare	24 991 (30.6)	—
Other	2493 (3.05)	—
Unknown	125 (0.15)	—

*Note:* Bold values indicate *P* Value < 0.05.

The average Age‐Adjusted Mortality Rate (AAMR) per 100,000 individuals was 11.08 (95% CI: 11.01–11.16), showing a clear upward trend. In 1999, the AAMR was 9.35 (95% CI: 8.99–9.72), rising significantly to 15.44 (95% CI: 15.06–15.82) by 2020. In 1999 and 2020, the secondary mortality rate due to peripheral circulatory complications among patients with type 2 diabetes was 1068 per 100,000 (10.58%) and 1802 per 100,000 (4.47%), respectively.

**FIGURE 1:**
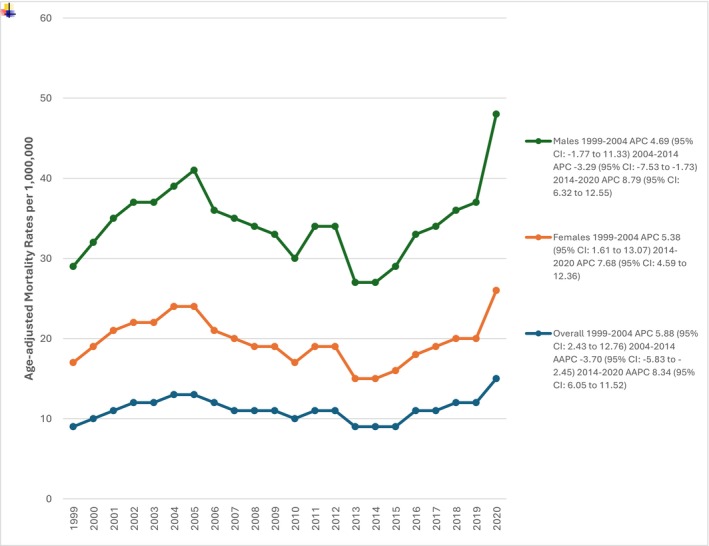
Overall and sex‐stratified PCC related AAMRs per 100,000 in type 2 diabetes mellitus patients in the United States, 1999 to 2020.

A closer look at the trends reveals distinct phases. From 1999 to 2004, there was a sharp increase in mortality, with an APC of 5.88 (95% CI: 2.43–12.76). However, between 2004 and 2014, the trend reversed, with a decline of −3.70 (95% CI: −5.83–−2.45). Unfortunately, from 2014 to 2020, the mortality rate surged again, with an APC of 8.34 (95% CI: 6.05–11.52), suggesting a concerning resurgence. (Figure 1).

### 3.2 Gender

Males exhibited higher vulnerability compared to females throughout the study period, with a consistently higher age‐adjusted mortality rate (AAMR). The AAMR in males was 14.89 (95% CI: 14.75–15.03), whereas in females, it was 8.35 (95% CI: 8.26–8.43). Over time, male mortality rates showed a significant increase from 12.07 (95% CI: 11.40–12.74) in 1999 to 21.60 (95% CI: 20.91–22.28) in 2020, while female rates declined from 10.69 (95% CI: 10.27–11.11) in 1999 to 7.65 (95% CI: 7.23–8.07) in 2020 (Table S2).

Examining annual percentage change (APC) trends revealed fluctuations over time. Among males, mortality increased from 1999 to 2005 (APC: 4.69; 95% CI: −1.77–11.33), followed by a decline until 2014 (APC: −3.29; 95% CI: −7.53–−1.73). However, a sharp upward trend was observed from 2014 to 2020 (APC: 8.79; 95% CI: 6.32–12.55). In females, an initial increase was seen from 1999 to 2004 (APC: 5.38; 95% CI: 1.61–13.07), followed by a decline until 2014, and then a subsequent rise until 2020 (APC: 7.67; 95% CI: 4.59–12.36) (Table S3).

These findings indicate a widening gender gap in mortality trends, with males experiencing a significant rise in mortality rates, particularly after 2014, while females showed a more stable or declining trend until recent years. The test for parallelism between males and females was statistically significant (*p* = < 0.05).

### 3.3 Age

From 1999 to 2020, the 85+ years age group had the highest mortality rate with a crude rate (CR) of 3945.93 per 1,000,000 population (95% CI: 3696.31–4195.56) and 21,452 total deaths. The annual CR in this group increased from 604 in 1999 to 1614 in 2020. The 45–54 years age group had the lowest crude mortality rate (CMR) at 63.41 per 1,000,000 (95% CI: 52.30–74.83). (Table S4).

### 3.4 Urbanisation

The AAMR was considerably higher in metropolitan and urban areas, with rates of 12.91 in small metropolitan areas, 11.72 in medium metropolitan areas, 10.07 in large central metropolitan areas, and 9.05 in large fringe metropolitan areas. In contrast, micropolitan and rural areas exhibited even higher mortality rates, with 13.75 in micropolitan areas and 18.62 in non‐core rural areas (Table S5).

**FIGURE 2:**
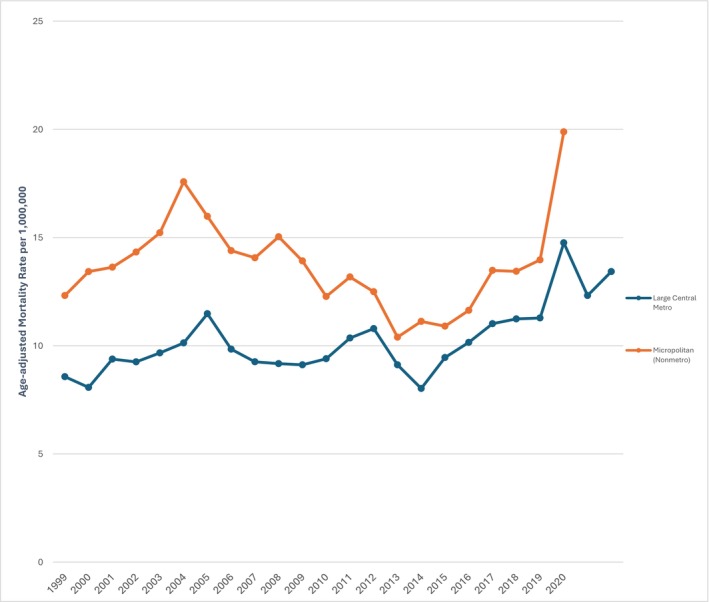
PCC‐related AAMRs in T2DM patients per 1,000,000 stratified by urbanisation per 1,000,000 in the US, 1999 to 2020.

From 1999 to 2016, the AAMR in large central metropolitan areas showed minimal change (APC = −0.22, 95% CI: −0.83–1.28), but this was followed by a steep upward trajectory until 2020 (APC = 9.16, 955 CI: 1.52–17.36) (Figure 2) The average APC over the two decades for large central metropolitan areas was recorded at 1.86 (95% CI = 0.33–3.41) (*p* = 0.01). In micropolitan areas, the mortality rate increased sharply from 1999 to 2004 (APC = 6.36, 95% CI: 0.95–12.05), followed by a substantial decline until 2015 (APC = −4.13, 95% CI: −5.80–−2.41), before rising steeply again until 2020 (APC = 11.40, 95% CI: 6.56–16.44) (*p* = 0.003) (Table S3).

The test for parallelism between micropolitan areas and large central metro areas was statistically significant (p = < 0.05).

### 3.5 Census Region

Geographically, the Midwest region had the highest AAMR 12.86 (95% CI: 12.69 to 13.03), followed by West 12.27 (95% CI: 12.09–12.44), South 10.93 (95% CI: 10.81–11.06), and Northeast 8.02 (95% CI: 7.88–8.17) (Table S6).

**FIGURE 3:**
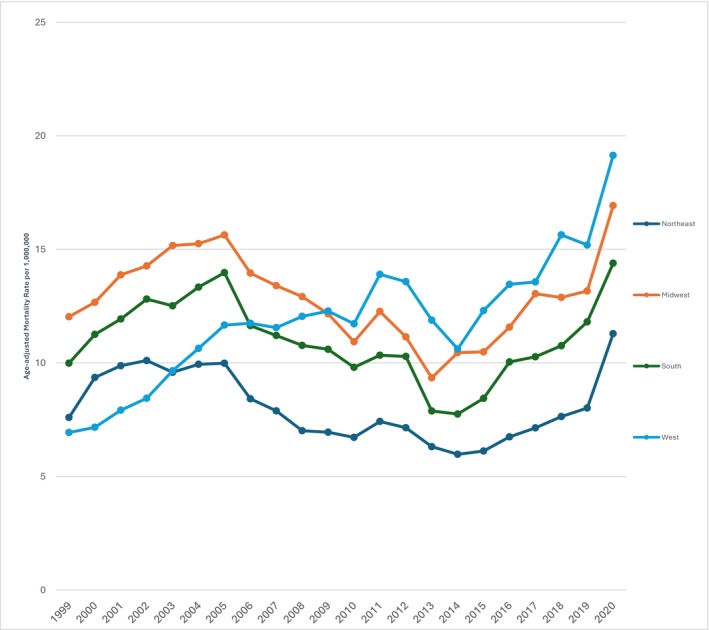
PCC‐related AAMRs in T2DM patients per 1,000,000 stratified by census region in the US, 1999 to 2020.

The trends in mortality varied across census regions over time. In the Northeast, there was a sharp increase in mortality from 1999 to 2001 (APC = 16.63, 95% CI: −9.46–50.26), followed by a significant decline from 2001 to 2015 (APC = −4.16, 95% CI: −6.37 to −1.92), and a subsequent increase from 2015 to 2020 (APC = 11.88, 95% CI: 3.87–17.67). In the Midwest, mortality initially decreased from 1999 to 2014 (APC = −4.57, 95% CI: −6.99 to −1.46), followed by an increase from 2014 to 2020 (APC = 8.52, 95% CI: 5.27–11.32). The South exhibited a steady rise in mortality trends across the decades, with significant increases observed from 1999 to 2013 (APC = 5.57, 95% CI: 3.10–8.33) and from 2013 to 2020 (APC = 8.96, 95% CI: 3.87–14.15) (Table S3). Similarly, in the West, mortality rose from 1999 to 2011 (APC = 5.34, 95% CI: 3.66–7.08), remained stable until 2013 (APC = −2.73, 95% CI: −8.60–3.59), and then sharply increased from 2013 to 2020 (APC = 8.71, 95% CI: 5.39–12.14) (Figure 3).

The test for parallelism showed significant differences in mortality trends between Northeast and Midwest (*p* = 0.003), Northeast and West (*p* = 0.0002), Midwest and West (*p* = 0.0002), and South and West (*p* = 0.0002), while no significant difference was found between Northeast and South (*p* = 0.182), indicating that mortality trends varied across most regions except between the Northeast and South.

### 3.6 State

**FIGURE 4:**
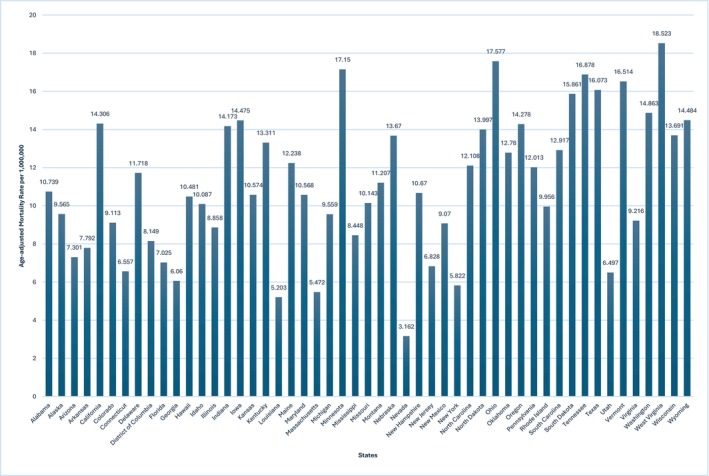
PCC‐related AAMRs in T2DM patients per 1,000,000 stratified by state in the US, 1999 to 2020.

There was a notable variation in AAMRs among states. West Virginia had the highest AAMR at 18.52 (95% CI: 17.35–19.70), with a total of 970 deaths, followed by Ohio with an AAMR of 17.58 (95% CI: 17.10–18.06) and Minnesota at 17.15 (95% CI: 16.43–17.87) per 1,000,000 US adults. In terms of absolute mortality burden, California reported the highest number of deaths, with 11,375 deaths recorded over the past two decades (Figure 4, Table S7).

### 3.7 Place of Death

From 1999 to 2020, PCC‐related deaths among T2DM patients in the United States varied based on place of death, where medical facilities accounted for 31,819 deaths (55.7%), nursing homes/long‐term care facilities for 24,991 deaths (43.8%), home settings for 19,523 deaths (34.2%), hospices for 2508 deaths (4.4%), and other locations for 2493 deaths (4.4%). Medical facility deaths peaked in 2020 (2002 deaths), as did nursing home deaths (1623 deaths). Home deaths increased from 379 in 1999 to 2275 in 2020. Hospice deaths, first recorded in 2004 (14 deaths), rose to 327 by 2020 (Table S8).

### 3.8 Race

American Indian or Alaskan natives showed the highest AAMR of 19.76 (95% CI: 18.45 to 21.06) followed by black or African American at 17.95 (95% CI: 17.62–18.28), Whites at 10.50 (95% CI: 10.43–10.58), and finally Asian or Pacific Islander at 6.11 (95% CI: 5.82–6.41) (Table S9).

**FIGURE 5:**
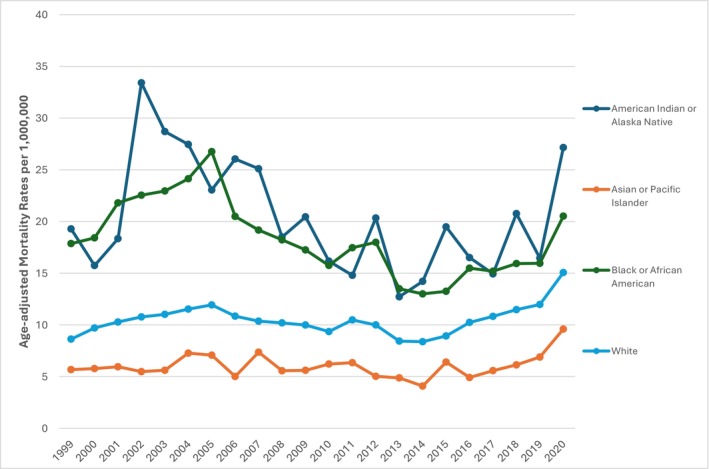
PCC‐related death in T2DM patients per 1,000,000 stratified by race in the US, 1999 to 2020.

Significant variations were observed across all races over the two decades. American Indians or Alaska Natives saw a steep rise from 1999 to 2002 (APC = 25.72, 95% CI: −11.82–79.24), followed by a steady decline until 2014 (APC = −5.99, 95% CI: −9.46 to −2.40), and finally an upward trajectory (APC = 8.53, 95% CI: 1.08–16.53). Black or African Americans followed a similar pattern, with a slight decline from 1999 to 2002 (APC = −1.15, 95% CI: −2.64–0.36), a decrease from 2004 to 2015 (APC = −5.62, 95% CI: −7.70 to −3.49), and an increase afterwards (APC = 8.07, 95% CI: 2.11–14.39). The White population also exhibited comparable trends, with an initial decline (APC = −2.63, 95% CI: −3.99 to −1.26), followed by an increase (APC = 10.58, 95% CI: 6.95–14.35). Asians and Pacific Islanders barely saw any change before experiencing an upward incline in 2017 (APC = 20.29, 95% CI: 4.37–38.64). (Figure 5).

The test for parallelism resulted in a *p* = 0.021, indicating a statistically significant difference in mortality trends across Black or African American and Asian and Pacific Islander groups. The parallelism test was rejected (*p* < 0.05) between the Black and white populations, indicating that mortality trends significantly differed among racial groups.

## 4 Discussion

Peripheral circulatory complications are a major health concern in patients with type 2 diabetes mellitus and are associated with a higher mortality risk due to cardiovascular events. Disparities exist in peripheral circulatory complications among patients with type 2 diabetes mellitus in the US state and are attributable to age, sex, socioeconomic status, ethnicity, and access to healthcare services. In this nationwide study using the CDC WONDER database, we observed a significant increase in the overall mortality rates attributed to peripheral circulatory complications in type 2 diabetes patients in the United States between 1999 and 2020. This may be attributable to the rising incidence of type 2 diabetes mellitus, poor disease management, lack of access to healthcare facilities, and increased prevalence of comorbidities among the general population [30].

Our study demonstrated that the crude mortality rates increased with age, with the highest rate observed in the 85+ year age group. This is in comparison to a study conducted in China, which demonstrated that the overall mortality among patients with type 2 diabetes mellitus was significantly higher among older individuals [31]. This may be attributable to the delayed onset and longer course of the disease among these individuals. Another study also showed that the crude mortality rate was highest in the 80–84 age group [32].

Gender disparities also exist among type 2 diabetes mellitus patients experiencing peripheral circulatory complications. We observed significantly higher mortality rates in males compared to females throughout the study period. This contrasts with a study by Yeo, J. L. et al. [33], which demonstrated that the overall incidence of cardiovascular disease was higher in men; however, the relative risk of developing peripheral circulatory complications was significantly greater in females, after controlling for possible confounding factors. Another study conducted in China demonstrated that males exhibit a higher disease burden than females, both in numbers and mortality rates [31]. Another study by Fatima M et al. [32], demonstrated that the overall age‐adjusted mortality rates were higher among males than females.

Sex hormones play a crucial role in the pathogenesis of diabetes and its cardiovascular complications, and both genders experience metabolic changes throughout their lives, especially females, who undergo more dramatic changes than males. However, gender disparities arise not only from genetic makeup but are also shaped by behavioural, cultural, and socioeconomic factors [34].

Geographic disparities in peripheral circulatory complications among type 2 diabetes mellitus patients were also studied in this pooled analysis. Our study showed that among the US states, the highest age‐adjusted mortality rate was observed for West Virginia. This is in comparison to the study conducted by Fatima M et al. [32], which showed that age‐adjusted mortality rates were higher in nonmetropolitan areas than in metropolitan ones and states like Ohio and West Virginia had higher rates. Similarly, Minnesota's mortality rate was 17.15 per 1,000,000 population. These elevated values may be attributed to two primary considerations. First, the comprehensive public health surveillance systems demonstrate particularly thorough case ascertainment compared to national standards. Second, the demographic composition of these states includes a proportionally larger elderly population.

The increasing mortality patterns, especially in underserved areas, may be caused by a number of systemic and structural causes in addition to demographic and geographic inequities. Access to vital vascular and diabetes care in isolated locations has been disproportionately impacted by rural hospital closures, which have resulted in inadequate management of peripheral problems and delayed diagnoses. Further aggravating results are opioid‐associated wound infections, which have been a rising issue in rural and socioeconomically disadvantaged communities, especially among those with diabetes and weakened immune systems. Additionally, the Special Diabetes Program for Indians (SDPI), a federal program created to offer prevention and treatment services to American Indian and Alaska Native communities, has demonstrated encouraging outcomes; nevertheless, its wider influence may be constrained by ongoing inequalities in funding and access.

Racial and ethnic disparities also exist in peripheral circulatory complications among type 2 diabetes mellitus patients. Our study demonstrated that the overall mortality rate was significantly higher among American Indians and Alaska Natives. These may be attributed to genetic makeup and socioeconomic disparities among these groups. A study conducted by Lopez J. et al. [35] showed that blacks and Hispanics exhibit a higher risk of developing diabetic cardiovascular complications. This study also demonstrated that white study participants had higher physical activity levels and functional capacity and demonstrated a lower risk of diabetic cardiovascular complications. In contrast to our finding, a study by Fatima M. et al. [32] demonstrated that the highest age‐adjusted mortality rate was among non‐Hispanic (NH) black individuals. These differences may stem from variations in the population included, the population characteristics, and statistical adjustments used.

By acknowledging these gender, racial, and ethnic disparities, healthcare providers and policymakers should work towards improving healthcare access, providing culturally tailored education, and implementing intervention strategies. Early detection and treatment of peripheral circulatory complications are imperative to enhance healthcare outcomes in patients with type 2 diabetes mellitus and a multidisciplinary approach should be adopted to address these concerns.

### 4.1 Strengths and Limitations

This study uses a nationally representative dataset from the CDC WONDER database spanning over two decades, allowing for robust trend analysis and stratification by key demographic variables such as race, sex, and geographic region. The use of Joinpoint regression improves the accuracy of temporal trend detection in mortality rates. However, the study is limited by the reliance on death certificate data, which may be subject to underreporting or misclassification of causes of death. Additionally, the database does not provide individual‐level clinical data such as disease severity, comorbidities, or access to care, which may confound observed disparities.

## 5 Conclusions

This study found an alarming rise in mortality from peripheral circulatory complications among diabetes patients in the United States between 1999 and 2020, with substantial differences by gender, age, race, and region. Men, older persons, rural residents, and American Indian/Alaska Native communities were disproportionately affected. While mortality initially decreased due to improved care, the recurrence after 2014 suggests the need for stronger interventions. To reduce unnecessary fatalities and close persisting disparities in outcomes, targeted public health policies, fair healthcare access, and early illness management must be implemented.

## Author Contributions

I.S. Conceptualization, Methodology, Software, Validation, Formal Analysis, Writing – Review and Editing, Visualisation, Project Administration. H.A. Screening, Data Curation, Writing – Original Draft. S.A. Data Extraction and Analysis, Software, Protocol Writing, Writing – Review and Editing. Q.M. Screening, Writing – Review and Editing, Risk of Bias Assessment, Graphical Abstract. A.I. Data Curation, Writing – Original Draft, Reviewing and Editing. S.R. Writing – Original Draft, Reviewing and Editing, Risk of Bias Assessment, Figures and Tables. S.S. Data Extraction, Risk of Bias Assessment, Writing – Original Draft. S.I.G. Data Extraction, Writing – Original Draft, Risk of Bias Assessment. M.F. Statistical Analysis, Manuscript Revision, Reference Management. M.F. Writing – Review and Editing, Manuscript Formatting. H.S. Literature Search, Clinical Interpretation, Quality Assurance. M.C. Supporting Informations Preparation, Data Verification, Proofreading. A.C. Supporting Informations Preparation, Data Verification, Proofreading.

## Ethics Statement

This study was exempted from the institutional review board's approval because it uses publicly available data that is de‐identified.

## Conflicts of Interest

The authors declare no conflicts of interest.

## Supporting information


Data S1.

## Data Availability

The data that supports the findings of this study are available in the Supporting Information of this article.
