# Supplementary material for: Disparities in Peripheral Circulatory Complication‐Related Mortality in Type 2 Diabetes Mellitus Patients: A CDC Analysis (1999–2020)
Source: Endocrinol Diabetes Metab. 2025 Jul 29;8(5):e70083. doi: 10.1002/edm2.70083 (PMC12307238; doi:10.1002/edm2.70083)
Supplement: Supplementary file 1 — Data S1. [file EDM2-8-e70083-s001.docx]

**Supplemental Table 1.** PCC–related Deaths in T2DM Patients, Stratified by Sex and Race, in the United States, 1999 to 2020

| **Deaths** | | | | | | | | | |  |
| --- | --- | --- | --- | --- | --- | --- | --- | --- | --- | --- |
| **Year** | **Overall** | **Women** | **Men** | **NH White** | **NH Black or African American** | **NH Asian or Pacific Islander** | **NH American Indian or Alaska Native** | **Hispanic or Latino** | **Population** | |
| 1999 | 2555 | 1267 | 1288 | 2099 | 399 | 34 | 23 | 408 | 279040168 | |
| 2000 | 2864 | 1439 | 1425 | 2385 | 419 | 38 | 22 | 443 | 281421906 | |
| 2001 | 3130 | 1625 | 1505 | 2559 | 505 | 39 | 27 | 475 | 284968955 | |
| 2002 | 3332 | 1704 | 1628 | 2717 | 531 | 38 | 46 | 518 | 287625193 | |
| 2003 | 3459 | 1681 | 1778 | 2818 | 552 | 46 | 43 | 587 | 290107933 | |
| 2004 | 3679 | 1863 | 1816 | 2987 | 590 | 61 | 41 | 604 | 292805298 | |
| 2005 | 3911 | 1885 | 2026 | 3134 | 672 | 65 | 40 | 702 | 295516599 | |
| 2006 | 3538 | 1638 | 1900 | 2903 | 539 | 48 | 48 | 706 | 298379912 | |
| 2007 | 3467 | 1586 | 1881 | 2826 | 522 | 74 | 45 | 772 | 301231207 | |
| 2008 | 3418 | 1493 | 1925 | 2824 | 501 | 53 | 40 | 869 | 304093966 | |
| 2009 | 3428 | 1513 | 1915 | 2819 | 499 | 66 | 44 | 881 | 306771529 | |
| 2010 | 3248 | 1435 | 1813 | 2679 | 460 | 72 | 37 | 991 | 308745538 | |
| 2011 | 3716 | 1616 | 2100 | 3079 | 523 | 79 | 35 | 1091 | 311591917 | |
| 2012 | 3688 | 1574 | 2114 | 3011 | 564 | 66 | 47 | 1301 | 313914040 | |
| 2013 | 3124 | 1346 | 1778 | 2586 | 433 | 71 | 34 | 1381 | 316128839 | |
| 2014 | 3148 | 1315 | 1833 | 2619 | 430 | 62 | 37 | 1568 | 318857056 | |
| 2015 | 3493 | 1403 | 2090 | 2860 | 472 | 104 | 57 | 1922 | 321418820 | |
| 2016 | 4035 | 1645 | 2390 | 3341 | 560 | 88 | 46 | 2133 | 323127513 | |
| 2017 | 4343 | 1793 | 2550 | 3616 | 576 | 104 | 47 | 2376 | 325719178 | |
| 2018 | 4721 | 1873 | 2848 | 3907 | 631 | 118 | 65 | 2627 | 327167434 | |
| 2019 | 5019 | 1916 | 3103 | 4173 | 648 | 141 | 57 | 2817 | 328239523 | |
| 2020 | 6477 | 2531 | 3946 | 5323 | 847 | 204 | 103 | 3525 | 329484123 | |
| **Total** | **81,793** | **36,141** | **45,652** | **67,265** | **11,873** | **1671** | **984** | **44,260** | **8,037,328,166** | |

**Supplemental Table 2** Overall and Sex-Stratified PCC-related Age-Adjusted Mortality Rates Per 1,000,000 in the United States, 1999 to 2020

| **Age-Adjusted Rate (95% CI)** | | | |
| --- | --- | --- | --- |
| **Year** | **Male** | **Female** | **Overall** |
| 1999 | 12.073 (11.401, 12.744) | 7.65 (7.226, 8.073) | 9.354 (8.991, 9.716) |
| 2000 | 13.157 (12.461, 13.853) | 8.53 (8.087, 8.974) | 10.371 (9.991, 10.751) |
| 2001 | 13.687 (12.983, 14.391) | 9.55 (9.083, 10.018) | 11.154 (10.763, 11.545) |
| 2002 | 14.593 (13.872, 15.315) | 9.915 (9.442, 10.389) | 11.705 (11.308,12.103) |
| 2003 | 15.466 (14.736, 16.197) | 9.652 (9.187, 10.116) | 11.955 (11.557, 12.354) |
| 2004 | 15.397 (14.677, 16.118) | 10.593 (10.109, 11.07) | 12.555 (12.148, 12.961) |
| 2005 | 16.719 (15.98, 17.459) | 10.594 (10.113, 11.076) | 13.089 (12.678, 13.5) |
| 2006 | 15.194 (14.501, 15.888) | 9.038 (8.597, 9.479) | 11.585 (11.202, 11.967) |
| 2007 | 14.738 (14.062, 15.413) | 8.632 (8.204, 9.061) | 11.145 (10.772, 11.517) |
| 2008 | 14.807 (14.136, 15.479) | 8.009 (7.559, 8.419) | 10.789 (10.426, 11.152) |
| 2009 | 14.225 (13.578, 14.872) | 7.963 (7.558, 8.369) | 10.566 (10.21, 10.922) |
| 2010 | 13.294 (12.672, 13.925) | 7.372 (6.986, 7.759) | 9.85 (9.509, 10.191) |
| 2011 | 14.869 (14.224, 15.515) | 8.139 (7.737, 8.542) | 10.966 (10.611, 11.322) |
| 2012 | 14.518 (13.889, 15.147) | 7.736 (7.348, 8.123) | 10.59 (10.245, 10.935) |
| 2013 | 11.824 (11.265, 12.383) | 6.467 (6.116, 6.819) | 8.769 (8.458, 9.08) |
| 2014 | 11.867 (11.314, 12.42) | 6.258 (5.913, 6.602) | 8.646 (8.34, 8.951) |
| 2015 | 12.997 (12.429, 13.564) | 6.505 (6.158, 6.851) | 9.303 (8.99, 9.615) |
| 2016 | 14.617 (14.02, 15.214) | 7.41 (7.046, 7.774) | 10.529 (10.2, 10.858) |
| 2017 | 15.093 (14.496, 15.69) | 7.939 (7.565, 8.312) | 11.034 (10.702, 11.367) |
| 2018 | 16.406 (15.793, 17.02) | 8.077 (7.706, 8.449) | 11.727 (11.388, 12.065) |
| 2019 | 17.309 (16.689, 17.928) | 8.192 (7.819, 8.564) | 12.158 (11.819, 12.498) |
| 2020 | 21.595 (20.91, 22.281) | 10.687 (10.265, 11.109) | 15.437 (15.057, 15.818) |
| **Total** | **11.083 (11.007, 11.6)** | **8.347 (8.261, 8.434)** | **11.083 (11.007, 11.16)** |

**Supplemental Table 3** Annual Percent Change (APC) of Peripheral Circulatory Complications - related Age-Adjusted Mortality Rates per 1,000,000 in the United States, 1999 to 2020

| **Year Interval** | **APC (95% CI)** |
| --- | --- |
| **Overall** | |
| 1999-2004 | 5.8840* (2.4257, 12.7604) |
| 2004-2014 | -3.7024* (-5.8378, -2.4512) |
| 2014-2020 | 8.3425* (6.0555, 11.5225) |
| **Male** | |
| 1999-2005 | 4.6877* (1.7736, 11.3398) |
| 2005-2014 | -3.2966* (-7.5281, -1.7331) |
| 2014-2020 | 8.7948* (6.3171, 12.5471) |
| **Female** | |
| 1999-2004 | 5.3797* (1.6111, 13.0721) |
| 2004-2014 | -5.0234* (-8.1400, -3.5987) |
| 2014- 2020 | 7.6699* (4.5965, 12.3589) |
| **American Indian or Alaskan Native** | |
| 1999-2002 | 25.7188 (-11.8192, 79.2365) |
| 2002-2014 | -5.9968* (-9.4624, -2.3985) |
| 2014-2020 | 8.5267* (1.0774, 16.5250) |
| **Asian or Pacific Islander** | |
| 1999-2017 | -1.1515 (-2.6426, 0.3624) |
| 2017-2020 | 20.2924* (4.3724, 38.6406) |
| **Black or African American** | |
| 1999-2004 | 6.1854 (-0.6951, 13.5427) |
| 2004-2015 | -5.6176* (-7.6981, -3.4903) |
| 2015-2020 | 8.0738* (2.1103, 14.3856) |
| **White** | |
| 1999-2004 | 5.1795* (0.7571, 9.7961) |
| 2004-2015 | -2.6341* (-3.9870, -1.2622) |
| 2015-2020 | 10.5832* (6.9436, 14.3466) |
| **Large Central Metro** | |
| 1999-2016 | 0.2186 (-0.8379, 1.2864) |
| 2016-2020 | 9.1567* (1.5238, 17.3634) |
| **Micropolitan (Nonmetro)** | |
| 1999-2004 | 6.3589* (0.9561, 12.0509) |
| 2004-2015 | -4.1265* (-5.8068, -2.4163) |
| 2015-2020 | 11.3982* (6.5695, 16.4457) |
| **Northeast** | |
| 1999-2001 | 16.6343 (-9.4649, 50.2573) |
| 2001-2015 | -4.1623* (-5.4024, -2.9060) |
| 2015-2020 | 11.8801* (6.3693, 17.6763) |
| **Midwest** | |
| 1999-2004 | 5.0798* (0.8699, 9.4653) |
| 2004-2014 | -4.5702* (-6.1485, -2.9653) |
| 2014-2020 | 8.2527* (5.2722, 11.3175) |
| **South** | |
| 1999-2004 | 5.5730* (0.5623, 10.8332) |
| 2004-2014 | -5.1531* (-6.9141, -3.3587) |
| 2014-2020 | 8.9686* (5.6726, 12.3675) |
| **West** | |
| 1999-2011 | 5.3414* (3.6275, 7.0837) |
| 2011-2014 | -7.9318 (-25.7394, 14.1461) |
| 2014-2020 | 8.7172* (5.3943, 12.1449) |

APC = Annual Percent Change, * Indicates that the annual percentage change (APC) is significantly different from zero at alpha = 0.05 level, AAMR = age-adjusted mortality rate.

| **Ten-Year Age Groups** | **Ten-Year Age Groups Code** | **Deaths** | **Population** | **Crude Rate** | **Crude Rate Lower 95% Confidence Interval** | **Crude Rate Upper 95% Confidence Interval** | **Crude Rate Standard Error** |
| --- | --- | --- | --- | --- | --- | --- | --- |
| 25-34 years | 25-34 | 21 | 9.2E+08 | 0.02 | 0.01 | 0.03 | 0 |
| 35-44 years | 35-44 | 199 | 9.31E+08 | 0.21 | 0.18 | 0.24 | 0.02 |
| 45-54 years | 45-54 | 1110 | 9.28E+08 | 1.2 | 1.13 | 1.27 | 0.04 |
| 55-64 years | 55-64 | 3794 | 7.66E+08 | 4.95 | 4.79 | 5.11 | 0.08 |
| 65-74 years | 65-74 | 7084 | 5.1E+08 | 13.88 | 13.55 | 14.2 | 0.16 |
| 75-84 years | 75-84 | 9972 | 2.99E+08 | 33.41 | 32.75 | 34.06 | 0.33 |
| 85+ years | 85+ | 7846 | 1.2E+08 | 65.65 | 64.2 | 67.1 | 0.74 |

**Supplemental Table 4** PCC-related Crude Mortality Rate (CMR) per 1,000,000 Stratified by Age Groups in the United States, 1999 to 2020

**Supplemental Table 5** PCC-related Age-Adjusted Mortality Rates per 1,000,000 Stratified by Urban-Rural Classification in the United States, 1999 to 2020

| **Year** | **Metropolitan** | **Nonmetropolitan** |
| --- | --- | --- |
| 1999 | 8.568 (7.911, 9.225) | 12.318 (11.031, 13.606) |
| 2000 | 8.073 (7.44, 8.707) | 13.425 (12.087, 14.763) |
| 2001 | 9.381 (8.702, 10.06) | 13.627 (12.286, 14.968) |
| 2002 | 9.252 (8.581, 9.923) | 14.326 (12.957, 15.695) |
| 2003 | 9.667 (8.985, 10.349) | 15.218 (13.815, 16.622) |
| 2004 | 10.128 (9.433, 10.824) | 17.575 (16.073, 19.077) |
| 2005 | 11.477 (10.742, 12.211) | 15.977 (14.553, 17.401) |
| 2006 | 9.839 (9.161, 10.517) | 14.393 (13.057, 15.728) |
| 2007 | 9.256 (8.605, 9.906) | 14.063 (12.749, 15.377) |
| 2008 | 9.171 (8.526, 9.817) | 15.034 (13.681, 16.388) |
| 2009 | 9.116 (8.48, 9.753) | 13.916 (12.632, 15.199) |
| 2010 | 9.397 (8.753, 10.04) | 12.275 (11.073, 13.477) |
| 2011 | 10.354 (9.687, 11.02) | 13.175 (11.942, 14.407) |
| 2012 | 10.791 (10.118, 11.464) | 12.493 (11.298, 13.688) |
| 2013 | 9.116 (8.505, 9.728) | 10.394 (9.311, 11.477) |
| 2014 | 8.024 (7.46, 8.588) | 11.123 (10.004, 12.241) |
| 2015 | 9.454 (8.846, 10.062) | 10.902 (9.818, 11.986) |
| 2016 | 10.153 (9.531, 10.774) | 11.633 (10.515, 12.751) |
| 2017 | 11.017 (10.381, 11.653) | 13.479 (12.287, 14.672) |
| 2018 | 11.238 (10.606, 11.871) | 13.438 (12.255, 14.622) |
| 2019 | 11.282 (10.653 ,11.91) | 13.966 (12.764, 15.167) |
| 2020 | 14.755 (14.043, 15.468) | 19.884 (18.471, 21.297) |
| **Total** | **10.068 (9.929, 10.207)** | **13.753 (13.483, 14.024)** |

**Supplemental Table 6** PCC-related Age-Adjusted Mortality Rates per 1,000,000, Stratified by Census Region in the United States, 1999 to 2020

| **Census Region** | **Year** | **Age-Adjusted Rate (95% CI)** |
| --- | --- | --- |
| Northeast | 1999 | 7.593 (6.884, 8.302) |
| Northeast | 2000 | 9.356 (8.573, 10.14) |
| Northeast | 2001 | 9.873 (9.073, 10.673) |
| Northeast | 2002 | 10.102 (9.298, 10.907) |
| Northeast | 2003 | 9.584 (8.804, 10.364) |
| Northeast | 2004 | 9.936 (9.143, 10.729) |
| Northeast | 2005 | 9.982 (9.191, 10.772) |
| Northeast | 2006 | 8.416 (7.695, 9.138) |
| Northeast | 2007 | 7.886 (7.189, 8.583) |
| Northeast | 2008 | 7.01 (6.358, 7.662) |
| Northeast | 2009 | 6.946 (6.301, 7.592) |
| Northeast | 2010 | 6.718 (6.084, 7.353) |
| Northeast | 2011 | 7.418 (6.755, 8.081) |
| Northeast | 2012 | 7.142 (6.507, 7.778) |
| Northeast | 2013 | 6.31 (5.71, 6.909) |
| Northeast | 2014 | 5.972 (5.392, 6.552) |
| Northeast | 2015 | 6.117 (5.537, 6.697) |
| Northeast | 2016 | 6.74 (6.131, 7.349) |
| Northeast | 2017 | 7.135 (6.518, 7.751) |
| Northeast | 2018 | 7.634 (7, 8.268) |
| Northeast | 2019 | 8.011 (7.372, 8.65) |
| Northeast | 2020 | 11.282 (10.521, 12.044) |
| **Northeast** | **Total** | **8.023 (7.877, 8.169)** |
| Midwest | 1999 | 12.031 (11.19, 12.872) |
| Midwest | 2000 | 12.665 (11.806, 13.524) |
| Midwest | 2001 | 13.874 (12.98, 14.769) |
| Midwest | 2002 | 14.267 (13.364, 15.17) |
| Midwest | 2003 | 15.164 (14.238, 16.089) |
| Midwest | 2004 | 15.245 (14.322, 16.168) |
| Midwest | 2005 | 15.633 (14.7, 16.565) |
| Midwest | 2006 | 13.95 (13.079, 14.822) |
| Midwest | 2007 | 13.398 (12.548, 14.247) |
| Midwest | 2008 | 12.916 (12.086, 13.746) |
| Midwest | 2009 | 12.159 (11.357, 12.962) |
| Midwest | 2010 | 10.928 (10.174, 11.681) |
| Midwest | 2011 | 12.261 (11.469, 13.053) |
| Midwest | 2012 | 11.141 (10.394, 11.888) |
| Midwest | 2013 | 9.341 (8.657, 10.024) |
| Midwest | 2014 | 10.449 (9.733l, 11.164) |
| Midwest | 2015 | 10.483 (9.773, 11.192) |
| Midwest | 2016 | 11.569 (10.829, 12.309) |
| Midwest | 2017 | 13.042 (12.263,13.821) |
| Midwest | 2018 | 12.878 (12.113, 13.642) |
| Midwest | 2019 | 13.164 (12.402, 13.925) |
| Midwest | 2020 | 16.921 (16.064, 17.779) |
| **Midwest** | **Total** | **12.859 (12.686, 13.032)** |
| South | 1999 | 9.985 (9.351, 10.62) |
| South | 2000 | 11.251 (10.582, 1.92) |
| South | 2001 | 11.929 (11.246, 12.612) |
| South | 2002 | 12.807 (12.104, 13.51) |
| South | 2003 | 12.51 (11.822, 13.198) |
| South | 2004 | 13.333 (12.628, 14.037) |
| South | 2005 | 13.971 (13.259, 14.683) |
| South | 2006 | 11.639 (10.999, 12.28) |
| South | 2007 | 11.203(10.58, 11.825) |
| South | 2008 | 10.767 (10.163, 11.371) |
| South | 2009 | 10.596 (10.003, 11.189) |
| South | 2010 | 9.801 (9.236, 10.365) |
| South | 2011 | 10.335 (9.763,10.906) |
| South | 2012 | 10.281 (9.719, 10.844) |
| South | 2013 | 7.881 (7.395, 8.366) |
| South | 2014 | 7.746 (7.271, 8.221) |
| South | 2015 | 8.435 (7.947, 8.923) |
| South | 2016 | 10.039 (9.514, 10.563) |
| South | 2017 | 10.267 (9.744, 10.791) |
| South | 2018 | 10.753 (10.226, 11.281) |
| South | 2019 | 11.804 (11.257, 12.351) |
| South | 2020 | 14.385 (13.788, 14.982) |
| **South** | **Total** | **10.932 (10.806, 11.058)** |
| West | 1999 | 6.931 (6.228, 7.634) |
| West | 2000 | 7.161 (6.452, 7.869) |
| West | 2001 | 7.914 (7.178, 8.65) |
| West | 2002 | 8.44 (7.689, 9.192) |
| West | 2003 | 9.654 (8.859, 10.45) |
| West | 2004 | 10.634 (9.808, 11.461) |
| West | 2005 | 11.663 (10.809, 12.518) |
| West | 2006 | 11.743 (10.896, 12.59) |
| West | 2007 | 11.55 (10.719, 12.381) |
| West | 2008 | 12.046 (11.205, 12.886) |
| West | 2009 | 12.278 (11.442, 13.113) |
| West | 2010 | 11.719 (10.908, 12.53) |
| West | 2011 | 13.897 (13.03, 14.764) |
| West | 2012 | 13.573 (12.729, 14.418) |
| West | 2013 | 11.88 (11.102, 12.657) |
| West | 2014 | 10.61 (9.884, 11.336) |
| West | 2015 | 12.302 (11.535, 13.07) |
| West | 2016 | 13.456 (12.664, 14.247) |
| West | 2017 | 13.561 (12.781, 14.341) |
| West | 2018 | 15.635 (14.806, 16.464) |
| West | 2019 | 15.189 (14.382,15.995) |
| West | 2020 | 19.134 (18.239, 20.029) |
| **West** | **Total** | **12.266 (12.092, 12.44)** |

**Supplemental Table 7** PCC-related Age-Adjusted Mortality Rates per 1,000,000 Stratified by States in the United States, 1999 to 2020

| **States** | **Age-Adjusted Rate (95% CI)** |
| --- | --- |
| Alabama | 10.739 (10.139, 11.339) |
| Alaska | 9.565 (7.607, 11.873) |
| Arizona | 7.301 (6.875, 7.727) |
| Arkansas | 7.792 (7.153, 8.431) |
| California | 14.306 (14.042, 14.571) |
| Colorado | 9.113 (8.513, 9.712) |
| Connecticut | 6.557 (6.04, 7.075) |
| Delaware | 11.718 (10.306, 13.13) |
| District of Columbia | 8.149 (6.588, 9.711) |
| Florida | 7.025 (6.809, 7.24) |
| Georgia | 6.06 (5.702, 6.418) |
| Hawaii | 10.481 (9.423, 11.538) |
| Idaho | 10.087 (9.024, 11.15) |
| Illinois | 8.858 (8.518, 9.198) |
| Indiana | 14.173 (13.575, 14.772) |
| Iowa | 14.475 (13.665, 15.285) |
| Kansas | 10.574 (9.811, 11.337) |
| Kentucky | 13.311 (12.6, 14.023) |
| Louisiana | 5.203 (4.757, 5.648) |
| Maine | 12.238 (11.123, 13.352) |
| Maryland | 10.568 (10.006, 11.13) |
| Massachusetts | 5.472 (5.121, 5.823) |
| Michigan | 9.559 (9.174, 9.944) |
| Minnesota | 17.15 (16.433, 17.867”) |
| Mississippi | 8.448 (7.753, 9.143) |
| Missouri | 10.143 (9.634, 10.653) |
| Montana | 11.207 (9.925, 12.489) |
| Nebraska | 13.67 (12.6, 14.739) |
| Nevada | 3.162 (2.694, 3.629) |
| New Hampshire | 10.67 (9.547, 11.792) |
| New Jersey | 6.828 (6.481, 7.175) |
| New Mexico | 9.07 (8.21, 9.931) |
| New York | 5.822 (5.607, 6.037) |
| North Carolina | 12.108 (11.645, 12.572) |
| North Dakota | 13.997 (12.29, 15.699) |
| Ohio | 17.577 (17.098, 18.056) |
| Oklahoma | 12.78 (12.039, 13.521) |
| Oregon | 14.278 13.525, 15.032) |
| Pennsylvania | 12.013 (11.656, 12.37) |
| Rhode Island | 9.956 (8.801, 11.11) |
| South Carolina | 12.917 (12.24, 13.594) |
| South Dakota | 15.861 (14.2, 17.522) |
| Tennessee | 16.878 (16.217, 17.538) |
| Texas | 16.073 (15.709, 16.436) |
| Utah | 6.497 (5.745, 7.248) |
| Vermont | 16.514 (14.541, 18.486) |
| Virginia | 9.216 (8.766, 9.665) |
| Washington | 14.863 (14.247, 15.479) |
| West Virginia | 18.523 (17.351, 19.695) |
| Wisconsin | 13.691 (13.086, 14.296) |
| Wyoming | 14.484 (12.347, 16.621) |

**Supplemental Table 8** PCC–related Deaths in T2DM Patients, Stratified by Place of Death in the United States, 1999 to 2024

| **Deaths** | | | | | |
| --- | --- | --- | --- | --- | --- |
| **Year** | **Medical Facility** | **Nursing Home/Long-term Care Facility** | **Hospices** | **Home** | **Other** |
|  |  |  |  |  |  |
| 1999 | 1225 | 876 | - | 379 | 62 |
| 2000 | 1364 | 1012 | - | 414 | 56 |
| 2001 | 1465 | 1118 | - | 445 | 87 |
| 2002 | 1558 | 1214 | - | 489 | 61 |
| 2003 | 1576 | 1184 | - | 585 | 97 |
| 2004 | 1647 | 1224 | 14 | 661 | 121 |
| 2005 | 1773 | 1337 | 33 | 663 | 96 |
| 2006 | 1532 | 1170 | 48 | 696 | 85 |
| 2007 | 1557 | 1067 | 60 | 684 | 96 |
| 2008 | 1236 | 1063 | 82 | 693 | 87 |
| 2009 | 1360 | 1002 | 113 | 808 | 81 |
| 2010 | 1314 | 963 | 100 | 760 | 109 |
| 2011 | 1469 | 1119 | 132 | 865 | 129 |
| 2012 | 1371 | 1059 | 149 | 989 | 119 |
| 2013 | 1101 | 925 | 117 | 849 | 128 |
| 2014 | 1120 | 966 | 125 | 840 | 93 |
| 2015 | 1226 | 1008 | 159 | 978 | 111 |
| 2016 | 1330 | 1146 | 239 | 1176 | 143 |
| 2017 | 1444 | 1191 | 272 | 1311 | 125 |
| 2018 | 1548 | 1355 | 246 | 1403 | 168 |
| 2019 | 1601 | 1369 | 292 | 1560 | 197 |
| 2020 | 2002 | 1623 | 327 | 2275 | 242 |
| **Total** | **31,819** | **24,991** | **2,508** | **19,523** | **2,493** |

**Supplemental Table 9** PCC-related Age-Adjusted Mortality Rates per 1,000,000 Stratified by Race in the United States, 1999 to 2020

| **Year** | **American Indian or Alaska Native** | **Asian or Pacific Islander** | **Black or African American** | **White** |
| --- | --- | --- | --- | --- |
| 1999 | 19.283 (11.779, 29.781) | 5.68 (3.885,8.019) | 17.863 (16.095, 19.631) | 8.625 (8.256, 8.994) |
| 2000 | 15.741 (9.615, 24.311) | 5.776 (4.023, 8.032) | 18.418 (16.638, 20.198) | 9.705 (9.316, 10.095) |
| 2001 | 18.34 (11.751, 27.288) | 5.954 (4.17, 8.243) | 21.802 (19.88, 23.723) | 10.281 (9.882, 10.679) |
| 2002 | 33.407 (23.973, 45.32) | 5.484 (3.82, 7.628) | 22.542 (20.602, 24.483) | 10.774 (10.369, 11.18) |
| 2003 | 28.697 (20.407, 39.23) | 5.612 (4.062, 7.56) | 22.951 (21.009, 24.893) | 11.019 (10.612, 11.426) |
| 2004 | 27.453 (19.33, 37.841) | 7.275 (5.496, 9.447) | 24.132 (22.155, 26.11) | 11.528 (11.114, 11.942) |
| 2005 | 23.051 (16.056, 32.059) | 7.075 (5.412, 9.088) | 26.754 (24.698, 28.809) | 11.934 (11.515, 12.353) |
| 2006 | 26.041 (18.687, 35.327) | 5.014 (3.657, 6.709) | 20.488 (18.723, 22.2520 | 10.846 (10.451, 11.242) |
| 2007 | 25.105 (17.935, 34.186) | 7.363 (5.74, 9.303) | 19.173 (17.493, 20.854) | 10.365 (9.981, 10.748) |
| 2008 | 18.501 (12.886, 25.73) | 5.567 (4.145, 7.32) | 18.225 (16.595, 19.854) | 10.195 (9.817, 10.573) |
| 2009 | 20.449 (14.471, 28.068) | 5.61 (4.291, 7.206) | 17.255 (15.703, 18.807) | 9.989 (9.619, 10.36) |
| 2010 | 16.165 (10.983, 22.945) | 6.217 (4.828, 7.881) | 15.751 (14.276, 17.227) | 9.359 99.002, 9.715) |
| 2011 | 14.8 (9.986, 21.128) | 6.349 (5.002, 7.946) | 17.461 (15.93, 18.993) | 10.482 (10.108, 10.855) |
| 2012 | 20.339 (14.659, 27.493) | 5.029 (3.873, 6.422) | 17.99 (16.467, 19.513) | 9.998 (9.637, 10.358) |
| 2013 | 12.724 (8.585, 18.164) | 4.877 (3.787, 6.183) | 13.497 (12.194, 14.8) | 8.436 (8.108, 8.765) |
| 2014 | 14.21 (9.782, 19.957) | 4.089 (3.12, 5.263) | 13.003 (11.741, 14.265) | 8.378 (8.053, 8.703) |
| 2015 | 19.484 (14.507, 25.617) | 6.404 (5.155, 7.654) | 13.249 (12.02, 14.478) | 8.931 (8.599, 9.262) |
| 2016 | 16.511 (11.9, 22.319) | 4.917 (3.927, 6.08) | 15.487 (14.168, 16.805) | 10.239 (9.887, 10.59) |
| 2017 | 14.935 (10.808, 20.117) | 5.579 (4.493, 6.664) | 15.192 (13.918, 16.467) | 10.829 (10.471, 11.187) |
| 2018 | 20.758 (15.841, 26.72) | 6.138 (5.016, 7.26) | 15.933 (14.656, 17.21) | 11.476 (11.112, 11.84) |
| 2019 | 16.455 (12.326, 21.523) | 6.896 (5.745, 8.046) | 15.958 (14.697, 17.219) | 11.975 (11.607, 12.342) |
| 2020 | 27.148 (21.676, 32.62) | 9.595 (8.263, 10.9270 | 20.516 (19.099, 21.933) | 15.063 (14.654, 15.473) |
| **Total** | **19.757 (18.449, 21.064)** | **6.114 (5.815, 6.413)** | **17.953 (17.623, 18.282)** | **10.504 (10.425, 10.584)** |

**Joinpoint settings:**

Joinpoint regression analysis was conducted using the Joinpoint Regression Program, version 4.5.0, developed by the Surveillance Research Program of the U.S. National Cancer Institute. The analysis employed a log-linear model to estimate trends in age-adjusted mortality rates over time. The minimum number of joinpoints was set to 0, and the maximum was limited to 3. Joinpoints were selected using the Monte Carlo permutation method with a significance level of 0.05. A total of 4,499 permutations were used to determine statistical significance. The grid search method was applied to identify the optimal number and location of joinpoints. Standard errors were derived from input data. Age-adjusted mortality rates were calculated using the 2000 U.S. standard population, and rates were expressed per 1,000,000 population. For each identified segment, the Annual Percent Change (APC) and its corresponding 95% confidence interval were calculated. A trend was considered statistically significant if the p-value was less than 0.05.

**WONDER Query Settings**

- ICD-10 code used: **E11.5** (Type 2 diabetes mellitus with circulatory complications)
- Dataset used: CDC WONDER – Multiple Cause of Death, 1999–2020
- Filters: U.S. population, age groups, place of death, race/ethnicity, census regions, urban-rural classification, etc.

**Disparities in Peripheral Circulatory Complication Related Mortality in Type 2 Diabetes Mellitus Patients: A CDC Analysis (1999-2020)**

STROBE Statement—Checklist of items that should be included in reports of ***cross-sectional studies***

|  | Item No | Recommendation | Page |
| --- | --- | --- | --- |
| **Title and abstract** | 1 | (*a*) Indicate the study’s design with a commonly used term in the title or the abstract | 1 |
|  |  | (*b*) Provide in the abstract an informative and balanced summary of what was done and what was found | 3 |
| Introduction | | |  |
| Background/rationale | 2 | Explain the scientific background and rationale for the investigation being reported | 3-4 |
| Objectives | 3 | State specific objectives, including any prespecified hypotheses | 4 |
| Methods | | |  |
| Study design | 4 | Present key elements of study design early in the paper | 5 |
| Setting | 5 | Describe the setting, locations, and relevant dates, including periods of recruitment, exposure, follow-up, and data collection | 5 |
| Participants | 6 | (*a*) Give the eligibility criteria, and the sources and methods of selection of participants | 5 |
| Variables | 7 | Clearly define all outcomes, exposures, predictors, potential confounders, and effect modifiers. Give diagnostic criteria, if applicable | - |
| Data sources/ measurement | 8* | For each variable of interest, give sources of data and details of methods of assessment (measurement). Describe comparability of assessment methods if there is more than one group | 5 |
| Bias | 9 | Describe any efforts to address potential sources of bias | - |
| Study size | 10 | Explain how the study size was arrived at | 5 |
| Quantitative variables | 11 | Explain how quantitative variables were handled in the analyses. If applicable, describe which groupings were chosen and why | 5 |
| Statistical methods | 12 | (*a*) Describe all statistical methods, including those used to control for confounding | 5 |
|  |  | (*b*) Describe any methods used to examine subgroups and interactions | 5 |
|  |  | (*c*) Explain how missing data were addressed | - |
|  |  | (*d*) If applicable, describe analytical methods taking account of sampling strategy | - |
|  |  | (*e*) Describe any sensitivity analyses | - |
| Results | | |  |
| Participants | 13* | (a) Report numbers of individuals at each stage of study—eg numbers potentially eligible, examined for eligibility, confirmed eligible, included in the study, completing follow-up, and analysed | 5  Supplemental Table 1  Figure 1 |
|  |  | (b) Give reasons for non-participation at each stage | - |
|  |  | (c) Consider use of a flow diagram | - |
| Descriptive data | 14* | (a) Give characteristics of study participants (eg demographic, clinical, social) and information on exposures and potential confounders | 5-8  Supplemental Table 2-9  Figure 2-5 |
|  |  | (b) Indicate number of participants with missing data for each variable of interest | - |
| Outcome data | 15* | Report numbers of outcome events or summary measures | 5-8 Supplemental Table 1-9  Figure 1-5 |
| Main results | 16 | (*a*) Give unadjusted estimates and, if applicable, confounder-adjusted estimates and their precision (eg, 95% confidence interval). Make clear which confounders were adjusted for and why they were included | 5-8  Supplemental Table 1-9  Figure 1-5 |
|  |  | (*b*) Report category boundaries when continuous variables were categorized | - |
|  |  | (*c*) If relevant, consider translating estimates of relative risk into absolute risk for a meaningful time period | - |
| Other analyses | 17 | Report other analyses done—eg analyses of subgroups and interactions, and sensitivity analyses | 5-8  Supplemental Table 2-9  Figure 2-5 |
| Discussion | | |  |
| Key results | 18 | Summarise key results with reference to study objectives | 8 |
| Limitations | 19 | Discuss limitations of the study, taking into account sources of potential bias or imprecision. Discuss both direction and magnitude of any potential bias | 9 |
| Interpretation | 20 | Give a cautious overall interpretation of results considering objectives, limitations, multiplicity of analyses, results from similar studies, and other relevant evidence | 9 |
| Generalisability | 21 | Discuss the generalisability (external validity) of the study results | - |
| Other information | | |  |
| Funding | 22 | Give the source of funding and the role of the funders for the present study and, if applicable, for the original study on which the present article is based | 9 |

*Give information separately for exposed and unexposed groups.

**Note:** An Explanation and Elaboration article discusses each checklist item and gives methodological background and published examples of transparent reporting. The STROBE checklist is best used in conjunction with this article (freely available on the Web sites of PLoS Medicine at http://www.plosmedicine.org/, Annals of Internal Medicine at http://www.annals.org/, and Epidemiology at http://www.epidem.com/). Information on the STROBE Initiative is available at www.strobe-statement.org.
